# Use of naproxen versus intracervical block for pain control during the 52-mg levonorgestrel-releasing intrauterine system insertion in young women: a multivariate analysis of a randomized controlled trial

**DOI:** 10.1186/s12905-021-01521-z

**Published:** 2021-10-29

**Authors:** Elaine Cristina Fontes de Oliveira, Thaís Baêta, Ana Paula Caldeira Brant, Agnaldo Silva-Filho, Ana Luiza Lunardi Rocha

**Affiliations:** Department of Obstetrics and Gynecology, Federal University of Minas Gerais, Avenida Professor Alfredo Balena 190, Santa Efigênia, Belo Horizonte, MG 30130100 Brazil

**Keywords:** Pain relief, Intrauterine contraception, Intrauterine device, Adolescent, Nulliparous, IUD insertion pain

## Abstract

**Background:**

To compare the effectiveness of 550 mg naproxen sodium versus 6 mL 2%-lidocaine intracervical block in pain lowering at the 52-mg levonorgestrel-releasing intrauterine system (LNG-IUS) placement in young women.

**Methods:**

In this randomized controlled trial, 100 women aged 15–24 years were block-randomized to receive either 6 mL 2%-lidocaine intracervical block 5 min before the LNG-IUS insertion or 550 mg naproxen 30 min before the procedure. Forty-nine women received 550 mg naproxen and 51 received intracervical block. The primary outcome was pain at LNG-IUS insertion. Secondary outcomes were ease of insertion, insertion failures, and correct IUS positioning. Neither participants nor doctors were blinded. Pain at insertion was assessed by using a Visual Analog Scale (VAS).

**Results:**

Women randomized to lidocaine intracervical block presented lower mean pain score at insertion, when compared to women who received naproxen (5.4 vs. 7.3, respectively; *p* < 0.001). Parous women had a 90.1% lower chance of experiencing severe pain (*p* = 0.004). There was a 49.8% reduction in the chance of severe pain for every 1-cm increase in the hysterometry (*p* = 0.002). The only complication observed during insertion was vasovagal-like reactions (7%). The insertion was performed without difficulty in 82% of the women. Participants in the intracervical block group presented higher proportion of malpositioned IUS on transvaginal ultrasound examination compared to women in naproxen group. Nevertheless, all the malpositioned IUS were inserted by resident physicians.

**Conclusion:**

Lidocaine intracervical block was found to be more effective than naproxen in reducing LNG-IUS insertion pain.

*Trial registration number*: RBR-68mmbp, Brazilian Registry of Clinical Trials, Retrospectively registered (August 4, 2020), URL of trial registry record: https://ensaiosclinicos.gov.br/rg/RBR-68mmbp/.

## Background

Unintended pregnancy is a serious global problem, accounting for more than half of all pregnancies in the world [1]. In Brazil, about 54% of conceptions are unplanned, with even higher rates in some high-risk groups, such as adolescents and young women [2]. Increased utilization of long-acting reversible contraceptive (LARC) methods is an important strategy to reduce unintended pregnancy rates, as LARC have higher efficacy, higher continuation rates, and higher satisfaction rates compared with short-acting contraceptives [3, 4].

Since LARCs require no effort after insertion to remain effective, efficacy with typical method use is similar to perfect use (0.2% failure rate) [5]. The US‐based Contraceptive CHOICE Project found LARC methods to be 20 times more effective than non-LARC methods, resulting in substantial reductions in teen pregnancy, birth, and abortion compared with national rates [4]. Both the American College of Gynecology and Obstetrics (ACOG) and the American Academy of Pediatrics (AAP) recommend LARC methods as the first-line contraceptive choice for preventing teenage pregnancy [6–9].

Although LARC methods, including intrauterine devices (IUD) and subdermal implant, are among the most cost-effective of all contraceptive methods they are still less commonly used than other methods [10–12]. In the United States (2011–2015), 99.4% of sexually active female teenagers had used some method of contraception. Nevertheless, only 5.8% of teenagers had ever used LARC, with 2.8% having used the IUD [12, 13]. The levonorgestrel-releasing intrauterine system (LNG-IUS) is a highly effective method with high rates of satisfaction and continuation in the first year of use [14, 15]. Nevertheless, fear of a painful placement is a common concern and still prevents some women from choosing the method. [16, 17]. Concern about insertion pain may also be a barrier for gynecologists to consider the IUD as a contraceptive option, especially for nulliparous women [18].

Several studies have evaluated different pain management strategies during IUD insertion, such as oral analgesia, cervical priming and local-anesthesia [19–23]. Nonetheless, the current evidence shows no consensus over an effective strategy. According to the 2015 Cochrane review, most NSAIDs, lidocaine gel, and misoprostol were not effective in reducing pain, although some lidocaine formulations, tramadol, and naproxen had some effect on reducing IUD insertion-related pain [24]. Recently, Samy et al. have also showed that vaginal dinoprostone was effective in reducing insertion pain in adolescents and young women [25].

This study aimed to compare the effectiveness of 550 mg naproxen sodium and 6 mL 2%-lidocaine intracervical block in pain relieving at the LNG-IUS placement in young women.

## Methods

The present research was conducted at the Family Planning Service, Department of Obstetrics and Gynecology, Hospital das Clínicas of Federal University of Minas Gerais (UFMG), Belo Horizonte, MG, Brazil. Its Ethical Committee approved the study, which was developed from March 2017 to August 2019. Participants were women who sought the Family Planning service for LNG-IUS placement for contraception or treatment of gynecological conditions. All women who agreed to participate in the study signed an Informed Consent Form (ICF). In the case of participants under 18 years old, both women and parents or legal guardian signed the ICF.

The study included nulliparous or parous women aged 15–24 years who were eligible for the LNG-IUS use, according to the World Health Organization (WHO) medical eligibility criteria for contraceptive use. Exclusion criteria were: uterine sounding less than 5 cm; cervical cytological abnormalities in the last 18 months; uterine cavity distortion (any congenital or acquired uterine abnormality distorting the uterine cavity in a manner that is incompatible with IUD insertion); current breast cancer, endometrial cancer or cervical cancer (awaiting treatment); recent history of pelvic inflammatory disease or untreated genitourinary tract infection; abnormal uterine bleeding of unknown cause; less than 6 weeks post-partum or post-abortion.

Women applying for use of LNG-IUS received family planning counseling and were asked to answer a questionnaire containing information on education level, parity, previous menstrual pattern, presence of dysmenorrhea, and previous use of contraception. Subsequently, a gynecologist collected the clinical history and performed a clinical examination.

Randomization was performed in block of five women each by the main researcher. Participants received a number according to the arrival order at the service. Then they were randomly drawn to one of two groups by cards stored in an envelope. Women were randomized to either 550 mg naproxen sodium 30 min before the LNG-IUS insertion or 6 ml 2%-lidocaine intracervical block 5 min before procedure. Forty-nine women received 550 mg naproxen and 51 received intracervical block. Neither participants nor doctors were blinded.

The 52 mg LNG-IUS (Mirena®—Bayer) placement was performed up to the 7th day of menstrual cycle by an obstetric gynecologist and/or a training resident physician, following the manufacturer's recommendations. The gynecologist performed the insertion if the resident was unable to insert the device. A urinary or blood pregnancy test was used to exclude pregnancy, if the woman was not using an effective contraceptive method. Intracervical block was performed prior to tenaculum placement using 6 ml of 2%-lidocaine distributed in a four-point technique, with 1.5 ml in each of the quadrants of the uterine cervix (at 1, 4, 7 and 10 o’clock).

After the LNG-IUS insertion, immediately after removing the speculum, each woman was presented with a 10 cm Visual Analog Scale (VAS) to quantify pain intensity during the whole procedure. VAS is a one-dimensional instrument containing a line numbered from zero to 10 and anchored on the ends by “no pain” and “worst imaginable pain”. Pain was classified as absent (0), mild (1–3), moderate (4–6), or severe (7–10).

Each insertion was classified as easy, difficult or failure. The need for ultrasound guidance was considered as a difficult insertion. After the procedure, the attending physicians completed a questionnaire with uterine sounding length, difficulty of insertion, need for ultrasound guidance, pain score and complications. A transvaginal ultrasound (TVUS) was performed to verify the LNG-IUS positioning 30 days after insertion, according to the service’s routine protocol. The LNG-IUS was considered malpositioned when described as partially expelled, rotated, embedded in the myometrium or located in the lower uterine segment or cervix.

The primary outcome was pain score after insertion for each group (Naproxen or intracervical block). Secondary outcomes were the following: ease of insertion, need for ultrasound guidance, insertion failures, complications and correct IUS positioning.

### Statistical analysis

Sample size was estimated using a two-sided test and assuming a SD of 28 mm, a VAS difference scores of 20 mm, an α of 0,05, and 95% power, which yielded a minimal sample of 42 participants per treatment group. Student’s t-test was used to compare two independent groups. The association between two categorical variables was performed using the Pearson’s Chi-square test. Fisher's exact test was used to compare groups as to the proportion of occurrence of a particular event of interest (categorical type variable). In the comparison between measurements performed in the same experimental unit or evaluated at two different moments, Student’s t-test for paired / dependent samples was used.

The association between each variable and pain (categorized as absent, mild or moderate vs severe) was assessed using a simple logistic regression model. Variables with *p* < 0.20 were included in a multiple model. Using the backward strategy, variables with *p* < 0.05 and the constant of significance were maintained in the final model. The quality of the adjustment was assessed using the Hosmer–Lemeshow test. The results were presented as odds ratios (OR) with respective 95% confidence intervals (95% CI). The association between qualitative variables and malpositioned IUD was assessed using Fisher's exact test. All statistical comparison with a *p* < 0.05 were assumed to be statistically significant.

## Results

We included 101 women considering the possibility of sample loss. One woman in the naproxen group had candidiasis and did not return for insertion after treatment (see Consort flowchart). One hundred women had the LNG-IUS inserted. Forty-nine women received 550 mg naproxen and 51 received intracervical block. There were no losses or exclusions after randomization. The participants in the two groups had comparable baseline sociodemographic and gynecological characteristics (Table 1)
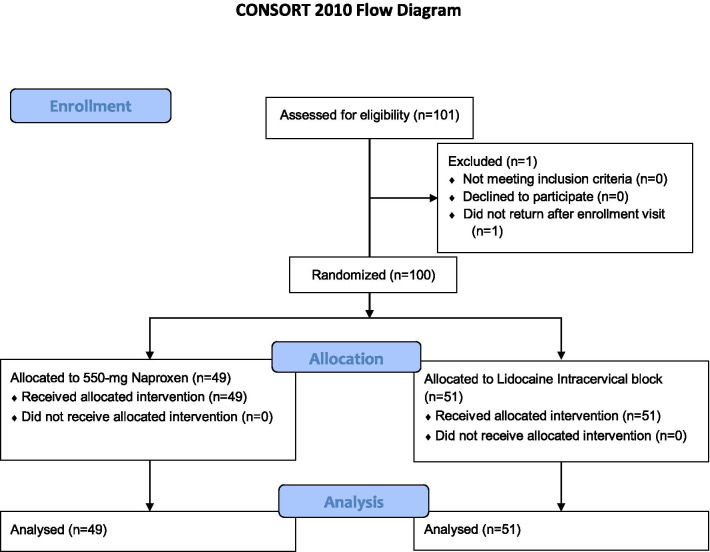
.

**Table 1:** Characteristics of participants

Variables	Naproxen	Block	*P*-value
Age	22.2 ± 2.0	22.2 ± 1.7	0.974
Race			0.438
White skin	30 (69.2)	35 (68.6)	
Skin of colour	19 (38.8)	16 (31.4)	
BMI (kg/m^2^)	23.2 ± 4.1	22.8 ± 3.6	0.228
Parity			0.498
Nulliparous	41 (83.7)	45 (88.2)	
Previous cesarean	1 (2.0)	0 (0.0)	
Previous vaginal delivery	7 (14.3)	5 (9.8)	
Any prior abortion	0 (0.0)	1 (2.0)	
Education			0.130
Less than high school degree	6 (12.2)	2 (3.9)	
High school degree	9 (18.4)	5 (9.8)	
Some college or higher	34 (69.4)	44 (86.3)	
Dysmenorrhea			0.806
Yes	19 (38.8)	21 (41.2)	
No	30 (61.2)	30 (58.8)	
Uterine sounding (cm)	7.24 ± 0.67	7.28 ± 0.81	0.758

The difficulty of insertion was statistically similar between the two groups. The only complication observed during the LNG-IUS insertion was vasovagal-like responses (such as dizziness, nausea and vomiting), which occurred in 7 women (7%), 3 women in the Naproxen group versus 4 women in the intracervical block group. Major complications such as uterine perforation or infection did not occur. No statistically significant association (*p* ≥ 0.05) was found between the pain relief method and complications. Resident physicians performed a total of 85 LNG-IUS insertions (41 in the naproxen group and 44 in the intracervical block group). Table 2 describes a comparison of insertion variables between the groups.

**Table 2:** Comparison of insertion variables between the naproxen group and the intracervical block group

Variable	Naproxen	Block	*P*-value
	N (%)	N (%)	
Difficulty of insertion			0.057
Ease	37 (75.5)	45 (88.2)	
Difficult	7 (14.3)	6 (11.8)	
Difficult, ultrasound guided	5 (10.2)	0 (0.0)	
Complications			1.000
No complications	46 (93.8)	47 (92.2)	
Vasovagal Reflex	3 (6.1)	4 (7.8)	
US after insertion			0.027
Normally positioned	47 (100.0)	45 (88.2)	
Malpositioned	0 (0.0)	6 (11.8)	
Health care provider			0.716
Resident	41 (83.7)	44 (86.3)	
Attending	8 (16.3)	7 (13.7)	

Women who received intracervical blockade for pain relieving presented higher rates of malpositioned LNG-IUS, compared to women in naproxen group (11.8% vs. 0%, respectively; *p* < 0.05). The LNG-IUS was found to be malpositioned in 6 women in the intracervical block group, even though all of these 6 insertions were considered easy by attending physicians. All malpositioned IUDs were inserted by resident physicians. Of the 6 malpositioned LNG-IUS, four were repositioned by ultrasound guidance. The remaining two IUDs were removed and a new device was inserted. Only one woman presented vasovagal response in this group and the remaining participants had no complications.

Women in the intracervical block group presented lower mean pain score, when compared to women in the naproxen group (5.4 ± 2.8 vs. 7.3 ± 2.1, respectively; *p* < 0.001). The two groups also presented a significant difference as to the ratings of absent or mild, moderate and severe pain (Table 3).

**Table 3:** Comparison of VAS pain scores between naproxen and intracervical block groups

VAS pain scores	Naproxen	Block	*P*-value
	(n = 49)	(n = 51)	
VAS pain			*p* < 0.001
Mean ± SD	7.3 ± 2.1	5.4 ± 2.8	
C.I	(6.7; 7.9)	(4.6; 6.2)	
Median	8.0 (5.0–9.0)	6.0 (3.0–8.0)	
Minimum–maximum	3.0–10.0	0.0–10.0	
Pain classification			*p* = 0.008
None or mild	3 (6.1)	15 (29.4)	
Moderate	13 (26.5)	13 (25.5)	
Severe	33 (67.4)	23 (45.1)	

Table 4 presents the factors associated with severe pain during the LNG-IUS insertion. The naproxen group was more likely to experience severe pain, when compared to the intracervical block group, in both univariate and multivariate analysis (OR 2.51, 95% CI 1.12–5.75, *p* = 0.026 and OR 3.67, 95% CI 1.48–9.65, *p* = 0.006; respectively). The factors associated with a lower chance of severe pain were as follows: previous pregnancy (non-nulliparous), non-white ethnicity and every 1-cm increase in the uterine sounding. The Hosmer–Lemeshow *p*-value indicates that the model is correctly specified.

**Table 4:** Factors associated with severe pain during the LNG-IUS insertion

Variables	Crude OR (95% CI)*	*p*-value	Adjusted OR (95% CI)**	*p*-value
Constant			3.190 (0.964; 13.713)	0.078
Pain relief method				
Block	–	–	–	–
Naproxen	2.511 (1.125; 5.755)	0.026	3.671 (1.488; 9.652)	0.006
Ethnicity				
White skin	–	–	–	–
Skin of colour	0.331 (0.087; 1.023)	0.079	0.243 (0.054; 0.864)	0.041
Education				
< high school graduation	–	–	–	–
High school graduation or equivalent	0.889 (0.142; 5.912)	0.899	–	–
≥ College graduation	2.022 (0.418; 10.855)	0.378	–	–
Attending physician				
Resident	–	–	–	–
Gynecologist	0.881 (0.291; 2.723)	0.822	–	–
Parity				
Nulliparous	–	–	–	–
Non-nulliparous	0.099 (0.015; 0.392)	0.004	0.068 (0.009; 0.300)	0.002
Uterine sounding (every 1-cm increase)	0.502 (0.272; 0.882)	0.021	–	–
Dysmenorrhea				
Yes	–	–	–	–
No	0.903 (0.400; 2.023)	0.805	–	–

*CI* Confidence interval, *OR* odds ratio, Hosmer–Lemeshow *p* = 0.957

*Crude OR refers to simple logistic regression; **adjusted OR refers to multiple logistic regression

Among the naproxen group, non-nulliparous women were less likely to experience severe pain in both univariate and multivariate analysis (OR 0.10, 95% CI 0.01–0.54, *p* = 0.013 and OR 0.07, 95% CI 0.007–0.46, *p* = 0.012; respectively). In this same group, the absence of previous dysmenorrhea was also associated with a lower chance of severe pain, in the multivariate analysis (OR 0.17, 95% CI 0.02–0.81, *p* = 0.04). The Hosmer–Lemeshow *p*-value (*p* = 0.998) indicates that the model is correctly specified. In the intracervical block group, there was no statistically significant association between the variables and severe pain.

## Discussion

Most IUD placements do not routinely require any pharmacological pain relief strategy. Nevertheless, some women experience substantial pain and the fear of pain during insertion continues to limit IUDs use especially in young women. Considering that pain experiencing is multifactorial and might be difficult to predict, several studies have identified predictors of pain, such as nulliparity, high level of education, not having had previous vaginal delivery, and history of dysmenorrhea [21, 23, 26–29]. These factors predicting pain should help health care professionals to identify women who would benefit from pharmacological interventions. The establishment of effective pain relief strategies during insertion could lead to a more widespread use of intrauterine devices.

A paracervical block with lidocaine is a commonly used part of analgesia in many outpatient gynecologic procedures. Lidocaine is the most common local anesthetic agent used because of low cost, stability, and low risk of allergic or adverse reactions [23]. Previous studies describe the use of different doses of lidocaine and different administration techniques (paracervical or intracervical block). We opted for a 6 mL 2%-lidocaine intracervical block based on the authors’ previous experience.

In this study, women submitted to lidocaine intracervical block presented significant lower pain scores, when compared to women who received naproxen prior to insertion. Pain during IUD placement is not confined to insertion, as the use of tenaculum, the uterine sounding, and the anesthetic injection itself can also contribute to an uncomfortable experience [26]. Therefore, the current evidences do not recommend the routinely use of intracervical block, although this procedure has been shown to reduce pain scores in previous studies [21, 22, 24–31].

A recent network meta-analysis has shown that lidocaine plus prilocaine (genital mucosal application) had the highest probability for being the most effective treatment in reducing pain at tenaculum placement, during IUD insertion and after IUD insertion, followed by lidocaine (paracervical). In this work, naproxen ranked as the least effective drug in reducing the pain at tenaculum placement [32].

This randomized controlled trial compared two different pain relief strategies that had previously been shown to have effect in reducing IUD insertion-related pain [24]. The results are important to encourage health care professionals to offer IUDs as a contraceptive option to adolescents and young women, as the insertion is generally considered easy, insertion-related complications are not common, and the pain can be managed in the outpatient clinic.

The study also assesses the factor associated with insertional pain: nulliparity, previous dysmenorrhea, health professional experience, hysterometry, ethnicity, and education level. Recognizing these factors predicting pain may help physicians to identify women who would benefit from pain relieving interventions.

The main limitation of the study is the lack of blinding. Neither participants nor doctors were blinded. The technical variability of professionals was also a limiting factor, as it might generate an information bias. The majority of the LNG-IUS was inserted by resident physicians, which might explain the higher pain scores in relation to those described in the published literature.

## Conclusion

The LNG-IUS is a first-line method of contraception for adolescents and young women. Considering that fear of pain during insertion might prevent some young women from choosing this method, a lidocaine intracervical blockade should be offered as a pain relief strategy, as it has been proven to be effective in reducing pain during the procedure.

## Data Availability

The data that support the findings of this study are available on request from the corresponding author, ALLR.

## Abbreviations

LNG-IUS: Levonorgestrel-releasing intrauterine system
IUS: Intrauterine system
VAS: Visual analog scale
LARC: Long-acting reversible contraceptive
ACOG: American College of Gynecology and Obstetrics
AAP: American Academy of Pediatrics
NSAIDs: Non-steroidal anti-inflammatory drugs
UFMG: Federal University of Minas Gerais
ICF: Informed consent form
WHO: World Health Organization
TVUS: Transvaginal ultrasound
SD: Standard deviation
